# About Pepsin Preparations

**Published:** 1874-05

**Authors:** 


### ABOUT PEPSIN PREPARATIONS.

For the cure of indigestion, various preparations of pepsin have lately been put in the market, as pepsin essence, elixir and pills. Those mixtures contain the effective ferment for digestion in more or less concentrated form. To the manufacture of it the stomachs of hogs, calfs and sheep are used. To test the value of a pepsin preparation, it is only necessary to mix with it blood fibrin in a glass tube, and immerse said tube into water of 86 deg. F., which, from a good pepsin preparation, gets dissolved at once.

